# Editorial: Advances in inflammatory bowel disease: Mucosal defense mechanisms against gut inflammation

**DOI:** 10.3389/fphar.2023.1147936

**Published:** 2023-02-21

**Authors:** Sinéad C. Corr, Gabriella Aviello

**Affiliations:** ^1^ Department of Microbiology, Moyne Institute of Preventative Medicine, School of Genetics and Microbiology, Trinity College Dublin, Dublin, Ireland; ^2^ Department of Pharmacy, School of Medicine, University of Naples Federico II, Naples, Italy

**Keywords:** Crohn’s disease, ulcerative colitis, mucosal immune defense, heat shock protein, epigenetics, caspase, phytochemicals

The delicate balance between immune tolerance and responsiveness in the intestine has its foundation in the synergistic action of multi-layered mucosal defense mechanisms regulating microbiota composition, epithelial integrity, and *lamina propria* immunity. In the last decades, there have been an increasing number of remarkable developments in understanding how mucosal mechanisms regulate intestinal homeostasis, powerfully contributing to the identification of new pharmacological strategies for the treatment of Crohn’s disease (CD) and ulcerative colitis (UC), collectively known as inflammatory bowel diseases (IBD). IBD are chronic and relapsing conditions, negatively affecting quality of life of both adult and pediatric patients. Thus, studying molecular targets able to clarify disease etiology and predisposition is crucial for early diagnosis, life-style modifications, and therapeutic interventions aimed at improving prognosis and minimizing life-threatening complications. This series of articles has been collected for the *Frontiers in Pharmacology* journal readership with the aim of showing some of the most recent developments in gut pathophysiology.

Smith and Creagh discuss the current literature in the area of caspase-4/-5/-11 expression and function in the gut and their role in the inflammation and pathogenesis of IBD (Smith and Creagh). In addition to the detailed description of how LPS activates caspase-4 and -5, the Authors also review important potential endogenous activators of non-canonical inflammasome signaling (oxPAPC, ER stress and cGAS/STING signaling) and their relevance to IBD and its pathogenesis. Despite the precise aspects of when and how the non-canonical inflammasome-associated caspases become functionally relevant during the development of IBD have yet to be clarified, it is concluded that their activation and signaling have the potential to provide new therapeutic strategies of clinical relevance in the management of IBD.

Another target particularly important in the context of inflammation is the heat shock (HS) family, composed of highly conserved molecules widely expressed in mammals and primarily upregulated under stressful conditions for the cell environment. Here, Gong et al. give an overview of the role of HS proteins and factors in maintaining gut mucosal homeostasis by regulating the survival and death of intestinal epithelial cells and promoting healing (Gong et al.). It is also emphasized the role of the HS family in the onset and development of colorectal cancer, of which chronic intestinal inflammation is a known risk factor. The findings discussed in this article highlight the importance of the HS family as a therapeutic target for inhibiting inflammation, sustaining mucosal repair, and potentially arresting the colitis-to-cancer progression. At the mechanistic level, in the research article by Liang et al. it has shown that heat shock transcription factor 2 (HSF2), highly expressed in UC, inhibits the activation of NLRP3 inflammasome of intestinal epithelial cells by regulating mitophagy (Liang et al.). Mitophagy is a biological process crucial for removing damaged mitochondria within the cell, and here the Authors suggest that the mechanism by which HSF2 promotes mitophagy involves the PARL/PINK1/Parkin signaling pathway.

Genome-wide association studies have classified >160 gene loci related to IBD thus far, however, only a small percentage of CD and UC patients present with these IBD-associated genetic variants, indicating that non-genetic factors also play an important role in altering gene expression levels and contributing to disease onset and progression. Xu et al. present a detailed review of DNA methylation, histone modification, and non-coding RNA mechanisms associated with IBD pathogenesis and how these epigenetic mechanisms affect T, B and intestinal epithelial cell function (Xu et al.). The Authors conclude the article by discussing how multi-omics analysis has revolutionized the study of biology and has improved our understanding of IBD by integrating unbiased large-scale data from different molecular levels.

Long-term therapies with current IBD medications may present loss of responsiveness or moderate to severe side effects, eventually affecting disease progression and patients’ quality of life. This underlines an unmet clinical need for new therapeutic strategies aimed at rendering prolonged IBD therapy more effective and safer. Moreover, chronic exposure to IBD medications, increasing prevalence of very-early onset IBD, and usual extra-intestinal manifestations require continuous developments in understanding the effectiveness and safety of novel compounds, including phytochemicals and nutraceuticals. Recent advances in computational methods and growing open access to clinical and experimental big data have rendered pharmacoinformatics an important and revolutionary tool for drug discovery and disease therapy planning. Using network pharmacology analysis and *in vivo* experimental validations, Liu et al. investigate core targets and pathways of *Pulsatilla* decoction (PD), a traditional Chinese medicine used to treat CD-like disease by suppressing inflammation and restoring gut epithelial barrier function (Liu et al.). On the other hand, using network pharmacology and *de novo* design, Tian et al. demonstrate how artemisinin, an antimalarial herbal medicine ingredient, can potentially be used for treating UC based on its capability to regulate 50 disease-related targets involved in biological processes, including reactive oxygen species metabolism, inflammation, proliferation and apoptosis (Tian et al.). Using a non-rodent model organism to study inflammation, Yang et al. dissect the effect of *Flos puerariae extract* (FPE) in a model of intestinal injury and show that FPE supplement maintains *Drosophila melanogaster*’s intestinal barrier function by regulating Nrf2-Keap1, JAK-STAT and Wnt signaling pathways (Yang et al.). IBD frequently affects extra-intestinal organs including the liver and growing evidence on the liver-gut axis suggest a potential association of chronic intestinal inflammation with certain liver diseases with various degrees of fibrosis. Liu et al. study the effect of tormentic acid in a rat model of liver fibrosis and, using transcriptomic and metabolomics analysis, find that this herbal ingredient ameliorates liver pathology by inhibiting the glycerophospholipid metabolism, the PI3K/Akt/mTOR, and the NF-κB signaling pathways (Liu et al.).

Our understanding of immune defense mechanisms at the intestinal barrier level and the discovery of promising new potential molecules for the treatment of IBD is not summarizable in one Research Topic, however, we believe that this article Research Topic contributes to an accessible, continue and in-depth comprehension of gut mucosal immunology.

